# An updated systematic review and meta-analysis of the prevalence of hepatitis B virus in Ethiopia

**DOI:** 10.1186/s12879-019-4486-1

**Published:** 2019-10-29

**Authors:** Teshiwal Deress Yazie, Mekonnen Girma Tebeje

**Affiliations:** Unit of Quality Assurance and Laboratory Management, School of Biomedical and Laboratory Sciences, College of Medicine and Health Sciences, University of Gondar, P.O. Box 196, Gondar, Ethiopia

**Keywords:** Hepatitis B virus, Viral hepatitis, Pooled prevalence, Meta-analysis, Ethiopia

## Abstract

**Background:**

Hepatitis B virus is one of the major public health concerns globally. It is highly infectious and can be transmitted from person to person through vertically or horizontally via contaminated body fluids. Despite the provision of an effective vaccine, it remains a major problem worldwide, particularly among the developing countries.

**Methods:**

Online electronic databases including PubMed, Google Scholar, Science Direct, African Index Medicus, African Journals Online, and WHO Afro Library were searched and published articles from 2010 to June 8, 2019, were considered. Both authors independently screened articles and extracted the data. Funnel-Plots and Egger’s test statistics were used to determine the presence of small-study effects and publication bias. The pooled prevalence of HBV was analyzed using the random-effects model. The possible sources of heterogeneity was analyzed through subgroup analysis, sensitivity analysis, and meta-regression.

**Results:**

The overall pooled prevalence of HBV was 6% and among subgroups, pregnant women, healthcare workers, and HIV positive patients accounted for 5% for each group. Relatively low prevalence (4%) was obtained among blood donors. The Egger’s test statistics (*p* = 0.747) indicated the absence of publication bias. In addition, from the sensitivity analysis, there was no influence on the overall effect estimate while removing a single study at a time. The level of heterogeneity was reduced among pregnant women, HIV positive and studies with unknown sampling techniques. After conducting meta-regression, province, study group, screening method, and quality of papers were identified as sources of heterogeneity.

**Conclusions:**

The overall pooled prevalence of HBV in Ethiopia was high. Strengthening and scaling up of the scope of the existing vaccination program and implementing novel approaches including screen-and-treat could be implemented to reduce the burden of the disease. Generally, the study can provide current prevalence estimate of HBV that could vital for intervention to tackle the disease.

## Background

Hepatitis B virus (HBV) is a major global public health problem [1]. The virus is highly infectious and can be transmitted through mother to child or via contaminated body fluid exposure such as unprotected sex, contaminated medical equipment, and blood donation [2, 3]. Despite the provision of an effective vaccine, it remains the main public health concern particularly among developing countries [4]. Hepatitis B virus infection can be determined by diagnosing the presence of hepatitis B surface antibody (HBsAb), hepatitis B pre-core antigen (HBeAg), hepatitis B pre-core antibody (HBeAb), hepatitis B surface antigen (HBsAg), or hepatitis B core antibody (HBcAb) sero-marker reactivity [5]. The presence of HBsAg represents active acute or chronic infections; whereas, the HBeAg indicates high viral replication while HBsAb and HBeAb are indications of HBV resolution [6]. In Africa, about 15 to 60% of the population is positive for at least one of the mentioned serological markers [7].

Hepatitis B virus is a life-threatening infection which attacks liver cells and can cause acute and chronic disease [8]. The acute HBV infection is usually a self-limiting disease [9]; however, chronic infection can cause liver cancer that becomes the second most common causes of death among cancer disease globally [10]. Annually more than 686,000 people die related to HBV infection complications, including hepatocellular carcinoma and liver cirrhosis [11]. Though HBV vaccination is the most important prevention mechanism [12], it is less likely accessible for private access due to the high cost of the vaccine [13].

Viral hepatitis causes high mortality and disability which is classified under the top 10 killer diseases [14]. About 2 billion people are infected with HBV globally from which over 240 million are chronically infected and are at high risk of developing liver cirrhosis and hepatocellular carcinoma [15]. According to the global burden of disease, an estimated 786,000 people die annually due to HBV infections from which 17, 43, and 40% were caused by acute infections, liver cancer, and cirrhosis, respectively [16]. The global prevalence of HBV infection is highly heterogeneous, and the highest prevalence (6.2 and 6.1%) is among the WHO Western Pacific and WHO African regions, respectively. As a part of the sub-Saharan region, Ethiopia is ranked medium to high endemicity for HBV infection [17]. Hepatitis B virus screening and case management are costly. In a high-prevalence area, HBV screening and treatment costs accounted for $29,230 per quality adjusted life-year [18]. Hepatitis B virus infection is also a major problem in Ethiopia with its prevalence vary from region to region even it is heterogeneous among studies within a region. The first systematic review and meta-analysis of viral infections in Ethiopia was conducted in 2016 [19]; however, the study included with 60% of old articles published before 2010. Due to this, it is difficult to estimate the current pooled prevalence estimate. In addition, as the study was conducted on more than one viral infections it lacked the detailed analysis of the HBV. Currently the Ethiopian government has given an emphasis to reduce the burden of HBV. Therefore, establishing the latest statistics regarding HBV pooled prevalence in Ethiopia could be vital in designing and implementing intervention programs and guidelines to reduce this national and international public health issue.

## Methods

### Study setting

This review was conducted in Ethiopia which is found in the Horn of Africa. Ethiopia is a highly populated developing country with a total population expected to be more than 100 million people within 1, 100,000 km^2^ landmass. The country is divided into nine administrative regions and two self-administrative cities. Recent studies indicated that HBV genotype A, D, C, E, and G are common in Ethiopia [20, 21]. The Federal Ministry of Health (FMoH) has established and implemented different strategic plans to reduce the burden of HBV in the country including the provision of selective vaccination programs for those high-risk groups such as health professionals, and those working in close contact with the local population and routine screening of patients suspected of viral hepatitis.

### Study design and protocol registration

The protocol of this systematic review and meta-analysis was designed according to the Preferred Reporting Items for Systematic Reviews and Meta-Analysis Protocols (PRISMA-P 2015) Guidelines [22]. The protocol was registered in the PROSPERO database with the protocol registration number of CRD42019131382.

### Article searching strategy

First, the PROSPERO database and database of abstracts of reviews of effects (DARE) (http://www.library.UCSF.edu) were searched to check whether published or ongoing projects exists related to the topic. The literature search strategy, selection of studies, data extraction, and result reporting were done in accordance with the Preferred Reporting Items for Systematic Reviews and Meta-Analyses (PRISMA) guidelines [23]. A comprehensive literature search was done in PubMed, Google Scholar, Science Direct, African Index Medicus, African Journals Online (AJOL), and WHO Afro Library Databases using keywords and Boolean operators (AND and OR) combination. The keywords used to search the mentioned databases were “hepatitis B virus”, “HBV”, “hepatitis B surface antigen”, “HBsAg”, “viral liver disease”, “viral hepatitis”, “prevalence”, “seroprevalence”, “seroepidemiology”, “magnitude”, “Ethiopia”, “year”. According to the databases’ requirement, the search string was customized for each database. As an example the PubMed search string of this review is attached as an additional file (Additional file 1). In addition, Google hand searching and screening of reference lists of the included and excluded studies were done. All full-text articles, published from 2010 to 2019, were considered and the last search was done on June 8, 2019.

### Eligibility criteria

Studies were eligible only if they were primary study full-text articles, published in peer-reviewed journals from 2010 to 2019, in English, and from the Ethiopian settings. In addition, articles which reported the magnitude of HBV prevalence were included. Studies conducted using valid HBV screening test methods, with clearly stated prevalence data or if missed the presence of sufficient data to calculate the prevalence were also considered as an inclusion criteria. Regarding the exclusion criteria, studies with unclear prevalence or methodological errors were not included in the study.

### Article selection and extraction

All the searched articles were imported into EndNote version X9 software and duplicate articles were removed. Then both authors screened articles independently to identify eligible studies according to the inclusion criteria. The data abstraction form was prepared in Microsoft Excel sheet which includes; the name of the first author, year of publication, year of study, setting (urban or rural), region, screening method (Enzyme-Linked Immunosorbent Assay (ELISA), rapid diagnostic tests (RDT), immunoassay (IA) or not mentioned), study group, sampling technique, sample size, study design, and number of HBV positive cases. Both authors extracted data files from the full-text articles independently. Any disagreement between the data extractors was resolved by consensus.

### Quality assessment

Both authors critically apprised the included studies independently using the Joanna Briggs Institute (JBI) quality assessment tool for prevalence studies [24]. The critical appraisal tool included nine parameters which have yes, no, unclear and not applicable options; 1) appropriate sampling frame, 2) proper sampling technique, 3) adequate sample size, 4) study subject and setting description, 5) sufficient data analysis, 6) use of valid methods for the identified conditions, 7) valid measurement for all participants, 8) using appropriate statistical analysis, and 9) adequate response rate. Operationally, a score of 1 was assigned for the yes response; whereas 0 scores was provided for no and unclear responses. Finally, the mean score was computed for each article. Then studies with below the mean score and “mean score and above” were categorized as poor and good quality, respectively [24]. The inter-rater agreement between the two data extractors was evaluated using Cohen’s Kappa. The result indicated that the inter-rater reliability coefficient (Kappa value) was 0.827 (*P* < 0.001) which is an indicator of excellent agreement.

### Data synthesis and analysis

Data were analyzed using metaprop program of STATA 15.1 software. When there is across study heterogeneity, the use of random-effects models is recommended [25]. In this case, the DerSimonian and Laird method is the most commonly used approach in meta-analysis [26]. The 퐼 *I*^2^ test statistics was used to check the presence of observed difference between-studies due to heterogeneity. Its value can range from 0 to 100% and the 75, 50, and 25% which represent the high, medium, and low heterogeneity among studies, respectively [27]. In addition, a *P*-value of < 0.05 was also used to the heterogeneity [28]. In this meta-analysis, the *I*^*2*^ value was high (97.77%) which is > 75% an indication of significant heterogeneity. Due to this reason, the analysis was conducted using a random-effects model at 95% CI as opposed to the fixed effects model to adjust the observed variability among studies. The sources of heterogeneity were analyzed using the sensitivity analysis, subgroup analysis, and meta-regression. Funnel plots and Egger’s test statistics were used to investigate publication bias and small-study effects. Data manipulation and analysis were done using STATA version 15.1 software (Stata Corp. LLC). College Station, Texas 77,845 USA for Windows.

## Results

### Study selection

**Fig. 1:**
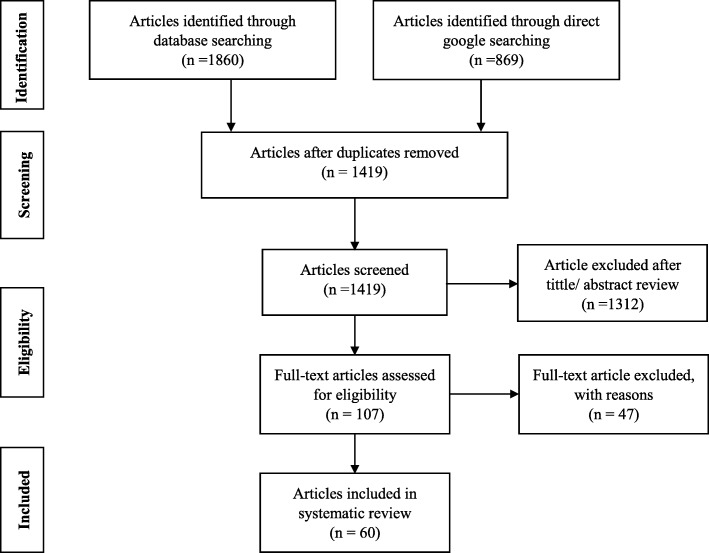
PRISA flow diagram for identification and selection of articles for inclusion in the review

Initially, a total of 2729 studies were identified from the databases and manual searching. From this, 1310 of the studies were removed due to duplication. The remaining 1419 articles were screened by their title and abstract and 1312 of the studies were excluded. Further, 107 full-text articles were refined and 47 of them were excluded due to being unrelated to the current study, studies on immigrants, review articles, studies conducted before 2010, and studies on immunization. Finally, a total of 60 [20, 29–87] studies were fulfilled the inclusion criteria and enrolled in the study [Fig. 1].

### Characteristics of included studies

A total of 60 articles were included in this systematic review and meta-analysis, with an overall sample size of 106,125 that conducted on the prevalence of HBV in Ethiopia. All the included studies were cross-sectional study designs and the most recent was conducted in 2019. Regarding regional coverage of HBV prevalence studies, more than half of the studies were obtained from Amhara region 22 (36.7%) [20, 29, 31, 36–38, 44, 46, 49, 50, 53, 58, 62, 63, 67–69, 71, 75, 78, 79, 82], Oromia region 12(20%) [32, 34, 41, 47, 48, 56, 60, 61, 64, 83, 85, 87], and Southern Nations, Nationalities and Peoples Region (SNNPR) 9(15%) [30, 43, 52, 65, 66, 70, 74, 76, 86]. The sample size across the studies was ranged from 108 [53] to 35,435 [44] obtained from the Amhara region. In addition, the qualities of each of the included studies was evaluated using the nine items risk of bias assessment tool (Table 1).

**Table 1:** Characteristics of the included studies in the systematic review and meta-analysis for the prevalence of hepatitis B virus in Ethiopia, 2019

First author, year	P. year	Region	Study group	Sampling technique	Sample	HBsAg +	D. method	Quality
Abate M., et al. [80]	2016	Somali	Blood donor	Entire sampling	2752	166	ELISA	Good
Abera B., et al. [79]	2017	Amhara	HIV positive children	Random sampling	253	5	ELISA	Good
Abera B., et al. [78]	2014	Amhara	Apparently healthy	Random sampling	481	15	RDT	Good
Akalu GT., et al. [77]	2016	SAC	Healthcare workers	Convenient sampling	313	55	IA	Poor
Amsalu A., et al. [76]	2018	SNNPR	Pregnant women	Consecutive sampling	475	34	ELISA	Good
Anagaw B., et al. [75]	2012	Amhara	Waste handlers	Unknown	200	6	RDT	Good
Asfaw MA., et al. [74]	2018	SNNPR	VCT	Random sampling	331	29	RDT	Good
Ataro Z., et al. [73]	2018	SAC	Blood donors	Entire sampling	6376	298	ELISA	Good
Ayele AG., et al. [72]	2013	SAC	Chronic liver diseases	Convenient sampling	120	43	RDT	Poor
Balew M., et al. [71]	2014	Amhara	HIV positive	Random sampling	395	24	RDT	Poor
Belayneh F., [70]	2015	SNNPR	HIV positive adult	Consecutive sampling	348	24	RDT	Good
Betela B., et al. [87]	2018	Oromia	General population	Random sampling	1343	146	RDT	Poor
Biadgo B., et al. [69]	2017	Amhara	Blood donors	Entire sampling	2294	121	ELISA	Good
Bialfew Y., et al. [68]	2018	Amhara	Blood donors	Consecutive sampling	403	19	ELISA	Good
Birku T., et al. [67]	2015	Amhara	Military personnel	Random sampling	403	17	RDT	Good
Bisetegen FS., et al. [66]	2016	SNNPR	Blood donors	Consecutive sampling	390	11	ELISA	Good
Chernet A., et al. [65]	2017	SNNPR	Pregnant women	Entire sampling	289	10	RDT	Good
Dabsu R., et al. [64]	2014	Oromia	Pregnant women	Convenient sampling	421	10	RDT	Good
Demsiss W., et al. [63]	2018	Amhara	Students	Random sampling	422	17	ELISA	Good
Deressa T., et al. [20]	2017	Amhara	HIV positive	Random sampling	308	17	PCR	Good
Deressa T., et al. [62]	2018	Amhara	Blood donors	Entire sampling	8460	102	ELISA	Poor
Desalegn Z., et al. [84]	2013	SAC	Healthcare workers	Convenient sampling	254	6	IA	Good
Desalegn Z., et al. [83]	2016	Oromia	Pregnant women	Entire sampling	202	11	ELISA	Good
Erena AN., et al. [61]	2014	Oromia	General population	Random sampling	353	26	IA	Good
G/micheal A., et al. [60]	2013	Oromia	Healthcare workers	Random sampling	220	9	RDT	Poor
G/egziabher D., et al. [59]	2016	SAC	General population	Entire sampling	482	102	RDT	Poor
G/mariam AA., et al. [58]	2019	Amhara	Healthcare professional	Entire sampling	332	15	RDT	Good
Habte Y., et al. [57]	2016	SAC	Blood donors	Entire sampling	4157	155	ELISA	Good
Hebo HJ., et al. [56]	2019	Oromia	Healthcare workers	Random sampling	240	6	ELISA	Good
Heyredin I., et al. [55]	2019	Mixed	Blood donors	Consecutive sampling	500	33	ELISA	Good
Kabato AA., et al. [86]	2016	SNNPR	Blood donors	Entire sampling	359	17	ELISA	Good
Kebede W., et al. [85]	2017	Oromia	Prisoners	Random sampling	156	9	ELISA	Good
Mekonnen A., et al. [54]	2015	SAC	Waste handlers	Random sampling	252	9	ELISA	Good
Mekonnen D., et al. [53]	2014	Amhara	Diabetes mellitus	*	108	4	RDT	Poor
Metaferia Y., et al. [52]	2016	SNNPR	Pregnant women	Convenient sampling	269	21	ELISA	Good
Mezgebo TA., et al. [81]	2018	Tigray	Pregnant women	*	328	18	IA	Good
Mohammed Y., et al. [51]	2016	Somali	Blood donors	Entire sampling	4224	460	ELISA	Good
Molla S., et al. [50]	2015	Amhara	Pregnant women	Random sampling	384	17	RDT	Good
Negash M., et al. [49]	2019	Amhara	Blood donors	Entire sampling	310	18	ELISA	Good
Negero A., et al. [48]	2011	Oromia	VCT	Entire sampling	384	22	RDT	Good
Schonfeld A., et al. [47]	2018	Oromia	Pregnant women	Consecutive sampling	580	31	RDT	Good
Seid M., et al. [46]	2014	Amhara	Pregnant women	Random sampling	385	19	ELISA	Good
Shiferaw E., et al. [44]	2019	Amhara	Blood donors	Entire sampling	35,435	230	ELISA	Good
Shiferaw Y., et al. [45]	2011	SAC	Waste Handlers	Random sampling	252	9	ELISA	Good
Shimelis T., et al. [43]	2017	SNNPR	HIV positive	*	477	30	RDT	Good
Shure W., et al. [42]	2018	SAC	Barbers	Convenient sampling	400	15	ELISA	Good
Taye S., et al. [41]	2014	Oromia	Chronic hepatitis	Entire sampling	358	80	RDT	Poor
Tegegne D., et al. [40]	2014	SAC	Pregnant women	*	265	8	ELISA	Good
Teklemariam Z., et al. [40]	2018	Harari	Blood donors	Entire sampling	4107	167	ELISA	Good
Tesfa H., et al. [38]	2013	Amhara	Clinically suspected	Entire sampling	2684	382	RDT	Good
Tessema B., et al. [37]	2010	Amhara	Blood donors	Entire sampling	6361	299	ELISA	Good
Tigabu A., et al. [36]	2019	Amhara	Blood donors	Entire sampling	5983	244	ELISA	Good
Tiruye G., et al. [35]	2018	Harari	Pregnant women	Random sampling	320	20	ELISA	Good
Umare A., et al. [34]	2016	Oromia	Pregnant women	Consecutive sampling	318	22	ELISA	Poor
Weldemhret L., et al. [33]	2016	Tigray	HIV positive	*	508	30	ELISA	Good
Wondimeneh Y., et al. [82]	2013	Amhara	HIV positive	Consecutive sampling	400	20	RDT	Good
Yami A., et al. [32]	2011	Oromia	Blood donors	Entire sampling	6063	126	ELISA	Good
Yizengaw E., et al. [31]	2018	Amhara	Healthcare workers	Random sampling	388	10	ELISA	Good
Yohanes T., et al. [30]	2016	SNNPR	Pregnant women	Random sampling	232	10	ELISA	Good
Zenebe Y., et al. [29]	2014	Amhara	Pregnant women	Random sampling	318	12	ELISA	Good

Key: *SAC* Self-Administrative City, *SNNPR* Southern Nations, Nationalities and Peoples’ Region, *ELISA* Enzyme-Linked Immunosorbent Assay, *RDT* Rapid Diagnostic Test, *IA* Immunoassay, *VCT* volunteer for counseling and testing

*: not stated

### Prevalence of HBV in Ethiopia

**Fig. 2:**
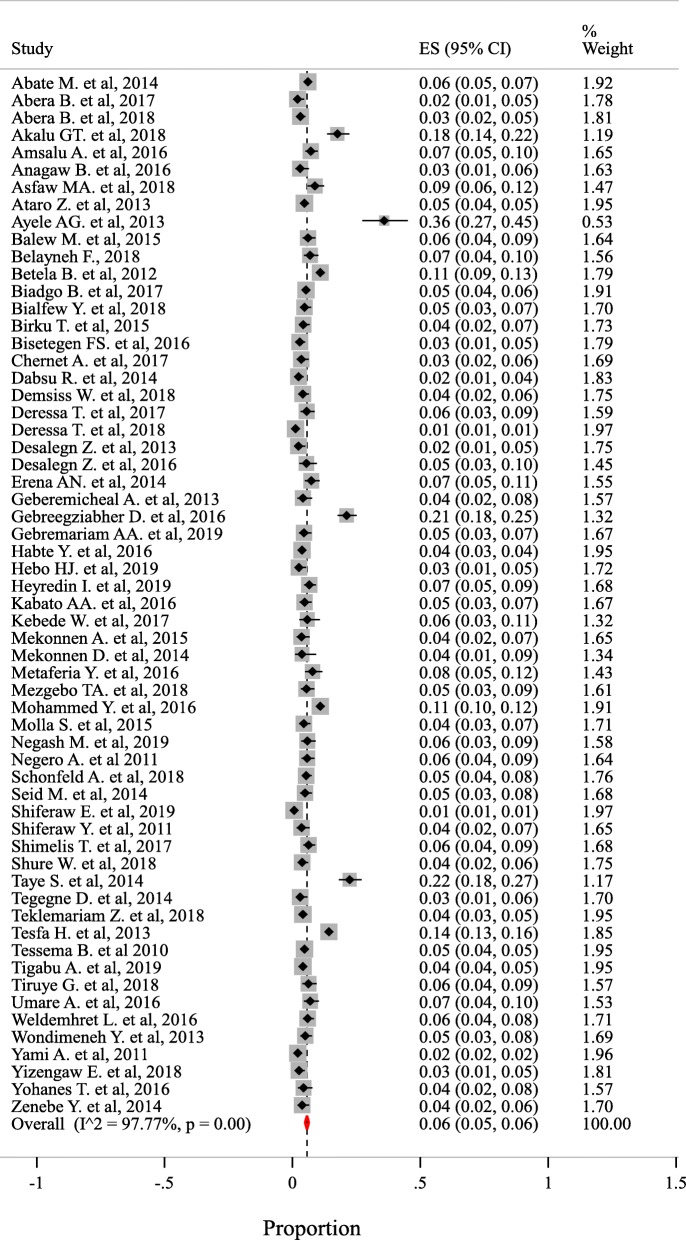
The pooled prevalence estimate of HBV in Ethiopia from 2010 to 2019

There was a wide HBV prevalence variation among included studies which is ranged from 1% in Amhara region to 36% in Addis Ababa city. Based on the random-effects model, the overall pooled prevalence among 106,125 was 6% (95% CI: 5 to 6%) with heterogeneity index (I^2^) of 97.77% (*p* < 0.001) (Fig. 2).

#### Subgroup analysis

Since this meta-analysis exhibited a considerable heterogeneity, subgroup analysis was done using study group, study quality, region/ city where the studies were conducted, year of publication, data collection year, sampling technique, screening method, and the setting was considered to identify the possible sources of heterogeneity among the studies. The subgroup analysis indicated that the heterogeneity level was slightly reduced among pregnant women (*I*^2^ = 52.2%), HIV positive study participants (*I*^2^ = 64.59%), studies conducted in SNNPR (*I*^2^ = 67.13%), studies conducted using probability sampling technique (*I*^2^ = 80.13%), and among studies those did not clearly indicated their sampling techniques (*I*^2^ = % 41.91). Geographically, the highest and the lowest prevalence of HBV were obtained from the Addis Ababa city 10% (95% CI: 6, 15%) and Amhara region 4% (95% CI: 3, 5%), respectively. Studies conducted on nonprobability sampling accounted for the highest 7% (95% CI: 5, 9%) followed by studies conducted through a survey. Concerning screening techniques, studies conducted using ELISA accounted for the least prevalence estimate of 4% (95% CI: 4, 5%) (Table 2).

**Table 2:** Subgroup analysis of the HBV pooled prevalence estimation in Ethiopia, 2019

Moderator variables	Variable category	Included studies	Prevalence % (95% CI)	I^2^%	% *P*-value
Study group	Blood donor	16	4 (0.03, 0.06)	98.99	0.00
	Pregnant women	14	5 (0.04, 0.06	52.20	0.01
	Healthcare worker	6	5 (0.05, 0.08)	89.47	0.00
	HIV positive	7	5 (0.04, 0.07)	64.59	0.01
	Others	17	9 (0.06, 0.11)	95.69	0.00
Study quality	Good quality	50	5 (0.04, 0.06)	97.74	0.00
	Poor quality	10	12 (0.07, 017)	98.12	0.00
Region/ city	Amhara	22	4 (0.03, 0.05)	97.95	0.00
	Oromia	12	6 (0.04, 0.09)	95.08	0.00
	SNNPR	9	6 (0.04, 0.07)	67.13	0.00
	Addis Ababa city	8	10 (0.06, 0.15)	96	0.00
	Others	9	6 (0.04, 0.07)	95.77	0.00
Year of publication	2010–2012	5	4 (0.02, 0.05)	94.44	0.00
	2013–2015	18	7 (0.05, 0.09)	94.85	0.00
	2016–2019	37	6 (0.05, 0.07)	98.02	0.00
Year of study	2010–2012	12	5 (0.03, 0.06)	92.28	0.00
	2013–2015	26	5 (0.05, 0.06)	85.59	0.00
	2016–2019	12	6 (0.04, 0.07)	89.54	0.00
Sampling techniques	Probability	20	5 (0.04, 0.06)	80.13	0.00
	Non-probability	14	7 (0.05, 0.09)	89.56	0.00
	Survey	20	6 (0.05, 0.07)	99.07	0.00
	Not stated	6	5 (0.03, 0.06)	41.91	0.105
Diagnosis method	ELISA	34	4 (0.04, 0.05)	97.98	0.00
	RDT	21	8 (0.06, 0.10)	95.07	0.00
	IA	4	8 (0.03, 0.13)	93.20	0.00
Setting	Urban	39	6 (0.05, 0.07)	98.41	0.00
	Mixed	21	5 (0.05, 0.06)	90.93	0.00

Key: *ELISA* Enzyme-Linked Immunosorbent Assay, *IA* Immunoassay, *RDT* Rapid Diagnostic Test; *SNNPR* Southern Nations, Nationalities and Peoples Region

#### Meta-regression and sensitivity analysis

A meta-regression analysis was done on the categorical variables including year of study, year of publication, study group, region, sample size, sampling technique, quality score, and screening methods. Among these variables, year of data collection was borderline significant. The remaining covariates including region/ city (*p* = 0.04), study group (*p* = 0.005), screening method (*p* = 0.017) and quality of papers (*p* = 0.001) were significantly associated with HBV pooled prevalence (Table 3). Sensitivity analysis was performed by removing a single study from the analysis in order to ensure the stability of the overall effect estimate. The result indicated that removing a single study from the analysis did not significantly influence the pooled estimate (Additional file 2).

**Table 3:** Meta-regression analysis of factors for the heterogeneity of HBV prevalence in Ethiopia, 2019

Moderator	Coefficient	Std. Error	*P*-value	Adjusted R^2^ (%)
Data collection year	.1547288	.0859943	0.077	4.07
Region/ city	.1328385	.0631128	0.040	5.98
Study group	.1623181	.0559312	0.005	13.81
Screening method	.3274201	.132906	0.017	9.54
Quality of papers	.8060166	.2361892	0.001	16.66

#### Publication bias and small study effects

**Fig. 3:**
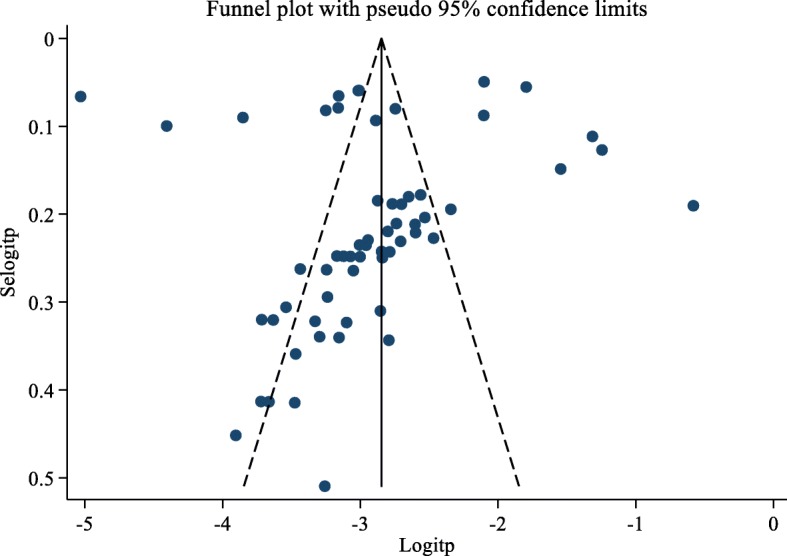
Funnel plot of the prevalence of HBV in Ethiopia from 2010 to 2019

The presence of publication bias was evaluated using funnel plots and Egger’s test. Each point in funnel plots represents a separate study and asymmetrical distribution indicates the presence of publication bias [5]. First, studies’ effect sizes were plotted against their standard errors and the visual evaluation of the funnel plot indicated no publication bias as the graph appear symmetrical (Fig. 3). The subjective evidence from the funnel plot was objectively confirmed using the Egger’s weighted regression statistics. According to the symmetry assumption, the *p-*value of 0.747 declares the absence of small study effects among the included studies.

## Discussion

Viral hepatitis is a serious disease which is caused by a number of hepatotropic viruses. Among these, HBV is by far the most clinically important pathogen that can cause both acute and chronic disease. Though there is recent scientific and clinical advancement of the anti-viral therapy, this pathogen is still a major public health concern globally, particularly among the developing countries [88, 89]. Hepatitis B virus prevalence is highly variable among different parts of the globe. Even it is inconsistent between studies within a country including Ethiopia. Therefore, in this review, we estimated the overall pooled prevalence of HBV among the Ethiopian population.

In the current study, all the included studies were cross-sectional and are therefore the indicators of the point prevalence of the disease. Considering this, HBV prevalence was ranged from 1 to 36% [62, 72] depending on the study population characteristics, study period, study design, and methods of laboratory screening. In most studies, the prevalence was lower than 8%, the threshold value of high transmission areas definition, [90, 91]. In this review, though the overall pooled prevalence estimate (6%) showed a slight decrement compared to the previous estimation (7.4%) [19], it is still a burden that represents an additional challenge for the national health system which is already fighting with the different infectious and noninfectious disease. In comparison to other studies conducted elsewhere, the result was more or less comparable to a finding from Thailand 5.1% [92]. However, it was significantly exceeded the global estimation (3.61%) and a finding from Iran (3%) [93, 94]. On the other hand, relatively far high estimates were obtained from Sudan 12.07%, Cameroon 10.6%, Burkina Faso 11.21%, Ghana 12.3%, Nigeria 13.6%, East Asia 8.6%, WHO Africa region 8.83%, and Somalia 19% [8, 93, 95–100]. The possible explanation for this great difference could be due to the fact that HBV might be hyperendemic in the mentioned countries. In addition, socio-cultural factors, level of awareness and infection prevention practice by the community, and the levels of stakeholder involvement in infection prevention could be considered as a possible factors for the high or low level of prevalence estimates.

Though the pooled prevalence (4%) of HBV among blood donors was significantly decreased compared to the previous estimate 8.4% [19], our study raised concerns on strict adherence of safe blood supply in Ethiopia as 1 in 25 (4%) blood donors might be infected with the virus. As a control strategy, if the system includes rigorous blood screening techniques before transfusion and sensitization of blood donors with recent potential risky behavior to avoid infective blood donation. The decreased pooled prevalence could be due to increased awareness of the community regarding infection prevention and increased attention and involvement of stakeholders. However, a lower prevalence estimate (2.03%) was obtained from the Eastern Mediterranean countries [101]. The current prevalence among blood donors was much lower than findings from Nigeria 14%, Ghana 11.75%, Burkina Faso 11.73%, and Cameroon 10.5% [96–99]. The possible explanation could be due to high endemicity of the virus in the said countries than Ethiopia.

Concerning HBV among pregnant women, a high (5%) prevalence was noted that require prompt screening of pregnant women during their antenatal care visit and provide proper treatment to decrease the rate of mother to child transmission of the virus. Though the result was far higher than recent findings from India 1.01% [102] and Iran (1.18 and 1.25%) conducted at different time periods [103, 104], it was significantly lower compared to the findings from Nigeria 14.1%, Cameroon 11.11%, Burkina Faso 9.8%, and Ghana 13.1% [96–99]. Most probability this variability could be due to the endemicity of the virus among the mentioned countries than Ethiopia or it could be due to the increased attention of the Ethiopian government in providing care and follow-up for the pregnant women which could significantly reduce the transmission and prevalence of HBV among the pregnant women.

Similarly, the pooled prevalence estimate among health workers was accounted for 5%. This high prevalence could be due to the fact that health professionals are frequently exposed to risky biological fluids that might be infected with the virus. The prevalence was however lower than a finding from Cameron 9.5% [96]. The possible explanation for this difference could be due to the difference in viral endemicity between the nations, awareness, potential exposure in risky medical procedures, over workload, and working hours per day could be possible predisposing factors that could be considered while analyzing the factors responsible for the difference in prevalence estimates. The current prevalence (7%) among HIV was exactly similar to the previous estimate of 7% [19]. However, the result was lower than findings from Nigeria 13.6%, Burkina Faso 12.61%, and Cameron 12.9% [96, 99, 105]. For this reason, viral endemicity, community awareness on infection prevention, and the role of stakeholders with respect to infection prevention and control could the potential factors. Though the current prevalence of HBV among HIV patients was considerably low compared to the findings from the aforementioned counties, an urgent measure should be implemented to manage this vulnerable group as they appear more at risk than the general population.

Though there was no influence on the overall effect estimate while removing a single study at a time from the analysis, we tried to assess the possible sources of variability through subgroup analysis and meta-regression. In the subgroup analysis, the level of heterogeneity was significantly reduced among studies conducted on pregnant women and HIV positive study participants. On the other hand, the *I*^*2*^ value did not significantly reduce among regional subgroup analysis. The highest (4%) and least (10%) prevalence estimates were obtained from the Amhara region and Addis Ababa city, respectively. The low prevalence estimate in the Amhara region could be due to better awareness of the community to HBV disease. Whereas, the high prevalence from the Addis Ababa city could be due to the fact that a study with high prevalence among chronic liver disease was included in the study that might affect the overall pooled prevalence estimate in that area. The heterogeneity level did not reduce among quality score subgroup analysis, but significantly high prevalence estimate was noted on those studies with poor quality that could be due to sampling bias among poor quality studies. Concerning screening methods, a low prevalence estimate was obtained among studies conducted with ELISA than studies conducted with RDT or IA screening techniques. This could be due to the high specificity of ELISA than RDT or IA screening techniques. Further, the possible sources of heterogeneity were analyzed through meta-regression and finally, province, study group, screening method, and quality of papers were identified as having a statistically significant association with HBV prevalence.

## Limitations

The current review has incurred several limitations which includes lack of study from the Benishangul-Gumuz and Afar regions. Even the number of studies obtained from the Tigray, Harari, and Somali regions were very limited. In addition, the inconsistency of screening methods might also be significantly contributed to the heterogeneity of the pooled estimate. Due to these reasons, the results of this systematic review and meta-analysis might compromise the overall pooled prevalence estimate of HBV in Ethiopia. Nevertheless, the current study provides an insight into the burden of HBV in Ethiopia and could promote the development of appropriate measures.

## Conclusions

The pooled prevalence of HBV was high in Ethiopia and it is a major public health threat. Since large numbers of recent studies were included in the review, it can clearly indicate the current prevalence and epidemiology of HBV in the country that could be valuable for policymakers. Therefore, strengthening the scope of the existing vaccination program and the establishment of new sensitive screening methods is highly recommended. In addition, it will be better if the existing infection prevention program is revised and target specific task force should be organized at different levels of health facilities in order to increase the awareness of the community. Control efforts should be scaled up countrywide and novel approaches including screen-and-treat could be implemented to reduce the burden of the disease in Ethiopia. Further political will and strong community awareness will be key to effectively tackling the burden of HBV problem in Ethiopia. Additional study should be conducted particularly in those regions where studies did not conduct so far to fully understand the dynamics of HBV burden in Ethiopia.

## Supplementary information

**Additional file 1.** PubMed search string.

**Additional file 2.** Sensitivity analysis of HBV prevalence in Ethiopia.

## Data Availability

All the data generated or analyzed are included in this manuscript and attached as an additional files.

## Abbreviations

DARE: Database of Abstracts of Reviews of Effects
ELISA: Enzyme-Linked Immunosorbent Assay
HBcAb: Hepatitis B Core Antibody
HBeAb: Hepatitis B Pre-core Antibody
HBeAg: Hepatitis B Pre-core Antigen
HBsAb: Hepatitis B Surface Antibody
HBsAg: Hepatitis B Surface Antigen
HBV: Hepatitis B Virus
HIV: Human Immunodeficiency Virus
IA: Immunoassay
JBI: Joanna Briggs Institute
PRISMA: Preferred Reporting Items for Systematic Reviews and Meta-Analyses
PRISMA-P: Preferred Reporting Items for Systematic Reviews and Meta-Analysis Protocols
RDT: Rapid Diagnostic Tests
SNNPR: Southern Nations, Nationalities and Peoples Region
VCT: Volunteer for Counseling and Testing
WHO: World Health Organization
