# Supplementary material for: An updated systematic review and meta-analysis of the prevalence of hepatitis B virus in Ethiopia
Source: BMC Infect Dis. 2019 Oct 29;19:917. doi: 10.1186/s12879-019-4486-1 (PMC6820955; doi:10.1186/s12879-019-4486-1)
Supplement: Supplementary file 1 — Additional file 1. PubMed search string. [file 12879_2019_4486_MOESM1_ESM.docx]

**PubMed search string**

“((hepatitis B [Mesh]) OR (HBV [Mesh]) OR (hepatitis surface antigen [Mesh])) OR (HbsAg [Mesh]) OR (viral liver disease [Mesh]) OR (viral hepatitis [Mesh]) AND (prevalence [Mesh]) OR (seroprevalence [Mesh]) OR (seroepidemiology [Mesh]) OR (frequency [Mesh]) OR (magnitude [Mesh]) AND (Ethiopia [Mesh]) AND (year [Mesh])”
