# Supplementary material for: An updated systematic review and meta-analysis of the prevalence of hepatitis B virus in Ethiopia
Source: BMC Infect Dis. 2019 Oct 29;19:917. doi: 10.1186/s12879-019-4486-1 (PMC6820955; doi:10.1186/s12879-019-4486-1)
Supplement: Supplementary file 2 [file 12879_2019_4486_MOESM2_ESM.docx]

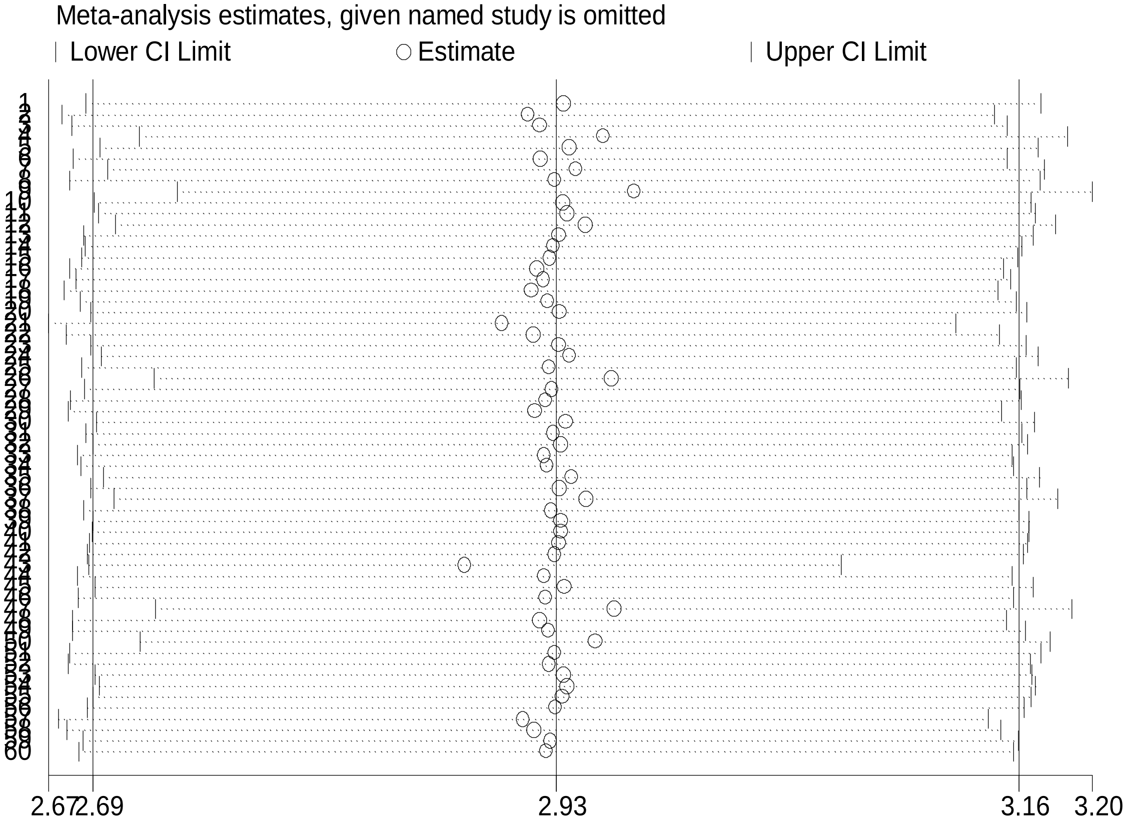

Meta-analysis estimates, given named study is omitted
Lower CI Limit
Estimate
Upper CI Limit
2.67
2.69
2.93
3.16
3.20
